# Impact of Magnetic Field Strength (1.5 T vs. 3.0 T) on the Prognostic Value of Quantitative ADC Indices in Patients After Out-of-Hospital Cardiac Arrest: A Prospective Multicenter Observational Study in Korea (The KORHN-PRO Registry)

**DOI:** 10.3390/jcm15145411

**Published:** 2026-07-10

**Authors:** Jae Hun Oh, Soo Hyun Kim, Seung Pill Choi, Se Won Oh, In Soo Cho, Yong Hwan Kim, Kyung Woon Jeung, on behalf of the Korean Hypothermia Network Investigators

**Affiliations:** 1 Department of Emergency Medicine, Eunpyeong St. Mary’s Hospital, College of Medicine, The Catholic University of Korea, Seoul 06591, Republic of Korea; emojh@catholic.ac.kr (J.H.O.); unidgirl@catholic.ac.kr (S.H.K.); 2 Department of Radiology, Eunpyeong St. Mary’s Hospital, College of Medicine, The Catholic University of Korea, Seoul 06591, Republic of Korea; oasis1979@gmail.com; 3 Department of Emergency Medicine, Hanil General Hospital, Seoul 01450, Republic of Korea; mensa@hanmail.net; 4 Department of Emergency Medicine, Samsung Changwon Hospital, Sungkyunkwan University School of Medicine, Changwon 16419, Republic of Korea; suka1212@hanmail.net; 5 Department of Emergency Medicine, Chonnam National University Hospital, Chonnam National University Medical School, Donggu, Gwangju 61469, Republic of Korea; neoneti@hanmail.net

**Keywords:** voxel ADC, cardiac arrest, outcome prediction, diffusion magnetic resonance

## Abstract

**Background/Objectives:** Although 3.0-Tesla (3.0 T) MRI is widely used and affects quantitative imaging, its impact on predicting outcomes for cardiac arrest survivors remains unexplored. This study investigated how magnetic field strength affects voxel-based apparent diffusion coefficient (ADC) analysis in resuscitated cardiac arrest patients. **Methods:** Using multicenter data from the Korean Hypothermia Network, ADC images were extracted from patients who underwent diffusion-weighted imaging. For each patient, the mean ADC and the percentage of voxels (PV) below specific thresholds were calculated. Receiver operating characteristic (ROC) analysis evaluated predictive performance for poor outcomes and established cut-off values. All parameters were compared between 1.5 T and 3.0 T MRI groups. **Results:** In the poor outcome group, mean ADC and PV indices showed no significant differences between the two magnetic strengths. However, in the good outcome group, the 3.0 T group exhibited significantly higher PV indices than the 1.5 T group. For predicting poor outcomes, 3.0 T MRI showed numerically higher area under the curve (AUC) values (0.877–0.895) than 1.5 T MRI (0.821–0.831). Cut-off values for predicting poor outcomes were also generally higher in the 3.0 T group. **Conclusions:** Magnetic field strength may affect quantitative ADC values and prognostic performance in cardiac arrest patients. Although AUCs were numerically higher at 3.0 T for all evaluated indices, this difference reached statistical significance only for PV500. Field strength should therefore be considered when establishing quantitative ADC thresholds.

## 1. Introduction

The pathophysiology of post-cardiac arrest brain injury (PCABI) is caused by ischemic and reperfusion injuries [1]. These two types of injuries increase patient mortality and can directly affect neurological recovery. Since there are no methods to directly treat neuronal damage that has already occurred, it is important to maintain sufficient cerebral perfusion and body temperature after the return of spontaneous circulation to minimize secondary brain injury. Both the American Heart Association and the European Resuscitation Council guidelines recommend temperature control as a treatment expected to improve neurological outcomes [2,3].

Patients who survive after cardiac arrest undergo evaluation for their neurological prognosis starting from the initiation of target temperature management (TTM). Among the various methods, MRI allows direct visualization of brain edema resulting from neuronal cytotoxic effects following PCABI, without being influenced by the administration of sedatives or neuromuscular blocking agents. Specifically, brain edema appears as hyperintensity on diffusion-weighted imaging (DWI) and as low signal intensity on the apparent diffusion coefficient (ADC) map. Owing to these characteristics, the quantitative measurement of the ADC is highly useful for predicting prognosis [4]. Voxel-based analysis enables quantification of the whole brain with only a few steps, making it easy to apply clinically. According to previous studies, the fraction of brain volume with an ADC below 650 × 10^−6^ mm^2^/s of 10% or more is highly correlated with a poor prognosis. Recent multicenter studies have also reported its utility in improving the predictive power for neurological outcomes [5,6,7,8].

However, the results of quantitative analysis are inevitably influenced by the device protocol [9]. In particular, compared with 1.5 T MRI, 3.0 T MRI provides a higher signal-to-noise ratio (SNR) and improved spatial resolution, and its superiority has been demonstrated in other diseases such as stroke [9,10]. Since brain injury after cardiac arrest has the unique characteristic of being a global ischemic injury, quantitative analysis of the whole brain may reveal even greater differences and exert a more sensitive influence on prognostic prediction [11,12]. Despite its potential significance, the impact of varying magnetic field strengths on the prognostic value of whole-brain ADC analysis in patients with cardiac arrest remains unknown.

Therefore, this study aims to evaluate the practical impact of various MRI magnetic field strengths (1.5 T vs. 3.0 T) used in real-world clinical settings on quantitative analysis values and prognostic predictive power, utilizing data from the Korean Hypothermia Network (KORHN), which includes more than 20 university hospitals and medical institutions [13].

## 2. Materials and Methods

### 2.1. Study Design and KORHN-PRO Registry

The KORHN-PRO 1.0 registry is a multicenter cohort involving 22 university hospitals across South Korea, and this study analyzed data collected from October 2015 to December 2018. Prior to initiating data collection, all the participating institutions engaged in discussions to standardize the TTM protocol, and the patient information collected was strictly managed through a dedicated research website. Registered patient information was assigned a new research number and anonymized during data extraction. The KORHN-PRO registry was approved by the Institutional Review Board of each institution and is registered at clinicaltrials.gov under protocol NCT02827422. Written informed consent was obtained from the legal representatives of all patients, and reconsent was obtained from the patients themselves if they regained consciousness.

This study included adult patients (aged 18 years or older) with out-of-hospital cardiac arrest (OHCA) of non-traumatic origin who remained in a coma after the return of spontaneous circulation and who received TTM. However, patients with a history of pre-arrest neurological impairment (Cerebral Performance Category [CPC] 3 or 4), intracranial hemorrhage (ICH), or brain surgery were excluded from the analysis.

### 2.2. MRI Acquisition and Quantitative Analysis

Eligibility was limited to patients whose MRI scans were performed 24–120 h post-ROSC. Specific exclusion criteria for the imaging data included non-DWI sequences, motion or technical artifacts, and poor resolution that precluded formal image analysis. Since the study design was not pre-specified at the time of registry enrollment, MRI machines and acquisition protocols were limited to the specific settings at each participating hospital.

As a multicenter registry study, this work included examinations acquired from scanners of multiple vendors (Philips, Siemens, and GE) and models using clinically established diffusion-weighted imaging protocols. Although our analysis focused on magnetic field strength (1.5 T vs. 3.0 T), field strength may inevitably correlate with scanner vendor and other technical factors that can influence quantitative ADC values, including scanner model, slice thickness, field of view, and acquisition matrix. These parameters are detailed in Supplementary Table S1. We did not harmonize the acquisition protocols across centers, nor did we statistically adjust for these system-level factors; therefore, the observed differences should be interpreted as the combined effect of magnetic field strength and its correlated acquisition parameters rather than because of magnetic field strength alone. Nevertheless, these findings highlight that quantitative ADC thresholds derived clinically from one system may not be directly transferable between scanners with different field strengths and protocols, and that scanner-specific or coordinated (harmonized) acquisitions are desirable before universal ADC cutoffs are applied.

DWI and ADC map images were stored in DICOM format after approval was obtained from each hospital. Collected DICOM files were anonymized using registry enrollment numbers after patient identifiers were removed, and the research team responsible for image analysis performed the analysis while blinded to the patients’ clinical information and outcomes.

**Figure 1:**
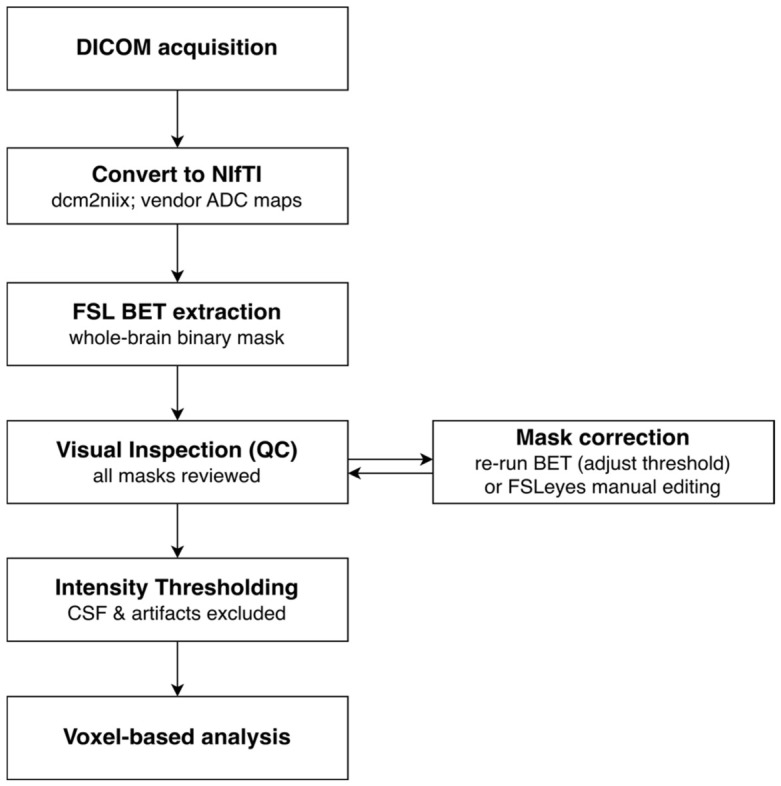
Schematic overview of the image-processing pipeline for voxel-based ADC analysis.

**Figure 2:**
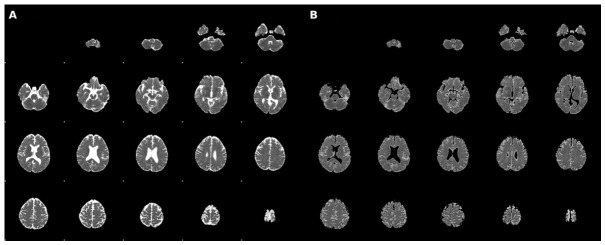
Representative whole-brain ADC masks shown as axial slices: (**A**) immediately after FSL BET extraction and (**B**) after exclusion of CSF and artifact voxels.

Quantitative voxel-based analysis of apparent diffusion coefficient (ADC) maps was performed over a whole-brain mask. Vendor-generated ADC maps (b = 0 and 1000 s/mm^2^) were converted to NIfTI format using dcm2niix. Brain extraction was performed using the Brain Extraction Tool (BET) of FMRIB software (FSL version 6.0.7 (release 2023), Oxford, UK) with a fractional intensity threshold of 0.4 and the robust (–R) option, yielding a whole-brain binary mask that included the cerebral hemispheres, deep gray matter, cerebellum, and brainstem, with no anatomical region excluded a priori. All masks were visually inspected slice-by-slice in FSLeyes by two board-certified neuroradiologists with 15 and 10 years of experience, respectively, both of whom were blinded to the clinical information and neurological outcomes throughout the entire image-processing and quality-control procedure. When a mask included non-brain tissue (residual skull, dura, or orbital structures) or excluded brain parenchyma, BET was re-run with an adjusted fractional intensity threshold, or the mask was manually corrected accordingly. Because all masks were reviewed under a strict quality-control standard, minor manual adjustments were applied in most cases. These adjustments were typically limited to small boundary voxels where BET slightly under or over extracted the brain margin. Extensive re-segmentation was rarely required, and no case relied on fully manual delineation. Studies with non-recoverable segmentation failure or non-correctable artifacts were excluded from the analysis. To minimize partial-volume contamination from cerebrospinal fluid (CSF), voxels with ADC values <50 or >1200 × 10^−6^ mm^2^/s were excluded from the final analysis mask: the lower threshold removed background and near-zero noise voxels, and the upper threshold removed CSF and CSF–parenchyma partial-volume voxels (free-water ADC ≈ 3000 × 10^−6^ mm^2^/s). The overall image-processing pipeline is summarized in Figure 1, and a representative example of the final analysis masks is shown in Figure 2.

The average ADC and percent-voxel (PV) indices were calculated for each patient, with the PV index defined as the proportion of voxels with ADC values below a specific threshold, as follows:

$$PVthreshold=\frac{Number\;of\;voxels\;ADC<threshold}{Total\;number\;of\;voxels\;in\;the\;brain\;mask}\times 100$$

The neurological outcomes of the patients were evaluated at 6 months after cardiac arrest using the CPC score. A poor outcome was defined as a CPC score of 3–5, whereas a good outcome was defined as a CPC score of 1–2.

### 2.3. Statistical Methods

Continuous variables were evaluated for normality using the Shapiro–Wilk test. Normally distributed continuous variables were expressed as the mean ± standard deviation, while non-normally distributed variables were presented as medians with interquartile ranges. Statistical differences between the two groups were evaluated using the t test or the Mann–Whitney U test. Categorical variables were presented as numbers and percentages and were analyzed using the chi–square test or Fisher’s exact test.

Receiver operating characteristic (ROC) curve analysis was employed to assess the ability to predict poor outcomes. The optimal cutoff values for maximizing sensitivity and specificity were determined using the Youden index, and cutoff values for false-positive rate (FPR) analysis were derived and compared at 100% specificity. The statistical significance of the differences in the area under the curve (AUC) between 1.5 T and 3.0 T MRI was verified using the DeLong test. A two-tailed *p*-value of less than 0.05 was considered to indicate statistical significance. All the statistical analyses were performed using SPSS version 23 (IBM Corp., Armonk, NY, USA) and MedCalc version 15.2.2 (MedCalc Software, Mariakerke, Belgium).

## 3. Results

### 3.1. Patient Demographics

**Figure 3:**
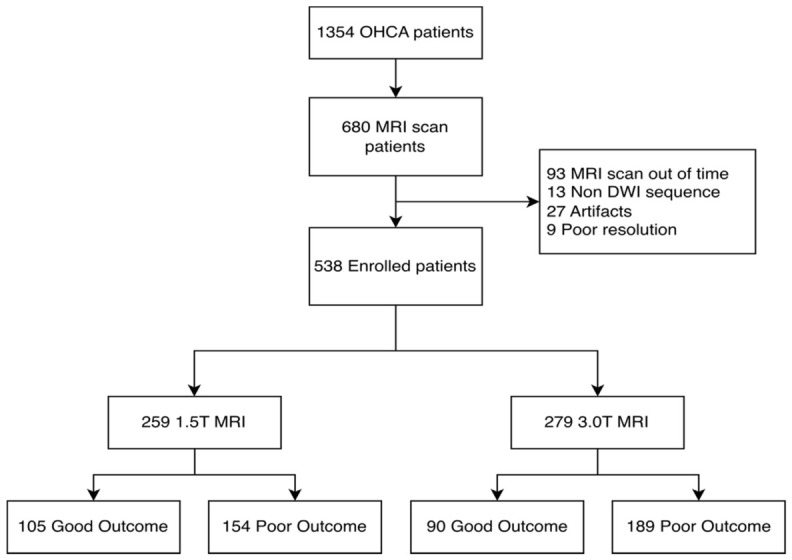
Flow diagram of the study population. OHCA—out of hospital cardiac arrest; MRI—magnetic resonance imaging; DWI—diffusion-weighted imaging.

During the study period, a total of 1354 patients who received TTM were enrolled from 22 participating hospitals. Among these patients, 680 underwent MRI scanning, and after excluding those who met the exclusion criteria, a final total of 538 patients were included in the analysis. Among the excluded examinations, nine were excluded because poor image resolution precluded satisfactory brain segmentation and formal image analysis (Figure 3).

Six months after ROSC, 195 patients (36.2%) demonstrated good neurologic outcomes, defined as a CPC score of 1–2. There were no significant differences in the mean age or sex distribution between the two groups. In terms of medical history, the prevalence of diabetes was greater in the 1.5 T group (26.3%) than in the 3.0 T group. Additionally, the proportions of patients with witnessed cardiac arrest, a cardiac etiology, and a favorable initial neurological status were greater in the 1.5 T group than in the 3.0 T group (Table 1).

### 3.2. Comparison of ADC Parameters by MRI Field Strength

The ADC analysis involved calculating the mean ADC value and the PV for each patient. We analyzed PV values from the PV500 to the PV650 in increments of 50 × 10^−6^ mm^2^/s. When comparing ADC indices according to MRI magnetic field strength within each prognostic group, no statistically significant differences were observed in the mean ADC or any PV indices within the poor outcome group. In contrast, within the good outcome group, the PV indices in the 3.0 T group were significantly greater than those in the 1.5 T group (Table 2).

### 3.3. Prognostic Performance and Optimal Thresholds

**Figure 4:**
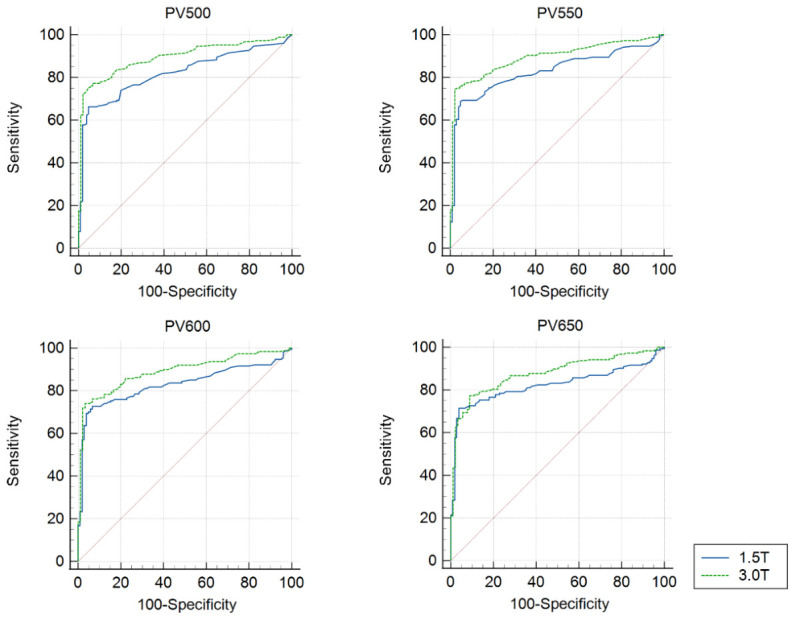
Comparison of independent ROC curves (1.5 T vs. 3.0 T).

ROC curve analysis was performed to determine the optimal PV cutoff values for predicting poor neurological outcomes. The area under the curve (AUC) for 1.5 T MRI ranged from 0.821 to 0.831, whereas 3.0 T MRI showed higher values, ranging from 0.877 to 0.895 (Figure 4). The cutoff values for predicting poor outcomes were also generally higher for 3.0 T MRI. For instance, at a false-positive rate (FPR) of 0%, the cutoff value for the PV650 was >72.0% with a sensitivity of 21.4% for 1.5 T MRI, while for 3.0 T MRI, a sensitivity of 21.2% was achieved at a cutoff of >79.8% (Table 3).

### 3.4. Comparison of ROC Curves

A direct comparison of the prognostic performance between the two magnetic field strengths revealed that the AUC of 3.0 T MRI at PV500 (0.895) was significantly higher than that of 1.5 T MRI (0.821; *p* = 0.025). For the PV550, the difference reached borderline significance (*p* = 0.051), whereas no statistically significant differences were observed for the other indices (Table 4).

## 4. Discussion

Neurological recovery in patients who achieve return of spontaneous circulation (ROSC) after cardiac arrest is a critical prognostic factor that affects their quality of life. To prevent premature withdrawal of life-sustaining treatment (WLST) and avoid the unnecessary depletion of medical resources, researchers have updated clinical guidelines and advanced studies on multimodal prognostic tools continuously. Building on previous attempts at quantitative imaging analysis, recent literature has increasingly emphasized the utility of voxel-based ADC analysis, which allows for a comprehensive evaluation of the whole brain [5,7]. While most studies have focused on its significance in predicting poor outcomes, some reports have also demonstrated robust performance in predicting favorable neurological outcomes [6].

However, a limitation of quantitative image analysis techniques is that factors such as the manufacturer and acquisition parameters of the equipment used in actual clinical practice vary, and these discrepancies can influence the results. Previous literature on CT attenuation differences in patients with cardiac arrest has also suggested that such variations must be considered [14,15]. In particular, as the clinical application of 3.0 T MRI has recently increased, several studies have analyzed the performance differences across various diseases according to MRI magnetic field strength [16,17,18].

This study holds significant value because it utilizes data from more than 20 hospitals, representing a broader scope than previous research on patients who recovered from out-of-hospital cardiac arrest (OHCA) and received TTM. The cohort includes major regional referral centers across South Korea, which ensures nationwide representativeness. Furthermore, a key strength of this study is that it remains free from the interference of a “self-fulfilling prophecy”, a phenomenon in which MRI results might influence treatment decisions. This is because the data were collected before established legal standards for the withdrawal of life-sustaining treatment were implemented in South Korea.

In this study, voxel-based mean ADC and PV indices of diffusion-weighted MRI showed a significant association with poor neurological outcomes. These results were consistent across both the 1.5 T and 3.0 T environments, demonstrating their utility for prognostic prediction in both settings. However, 3.0 T MRI yielded numerically higher AUCs across all threshold indices. These findings suggest that the higher signal-to-noise ratio (SNR) and improved spatial resolution of high-field MRI can more sensitively detect subtle ADC reductions caused by diffuse hypoxic brain injury. Previous research comparing MRI field strengths in acute stroke has reported that compared with 1.5 T, high-resolution 3.0 T MRI enhances the visibility of ischemic lesions. This enhancement, attributed to increased SNR and spatial resolution, suggests that higher field strength may likewise improve the detection of diffuse hypoxic brain injury. Consistent with this, multivendor and multi-institutional studies have shown that absolute ADC values vary substantially with field strength, vendor, and coil configuration [19]. In our data, however, this translated into a statistically significant gain in prognostic performance only for the PV500 index (*p* = 0.025), whereas the remaining indices showed numerically higher but non-significant AUCs.

This higher AUC at the PV500 index may indicate that the improved SNR of 3.0 T allows more precise quantification even at strict thresholds, by reducing signal distortion in low-ADC regions. These findings further imply that optimal cutoff values for prognostic prediction may need to be established separately across MRI field strengths in future multicenter studies or clinical guidelines [20]. Furthermore, they suggest that the accuracy of quantitative analysis may continue to improve as high-resolution MRI becomes more widely available. In conclusion, our findings suggest that quantitative ADC values and, for the PV500 index, prognostic performance may differ across MRI field strengths, providing a basis for developing field-strength-specific prognostic criteria.

This study has several limitations that should be considered. First, as a multicenter registry study, examinations were acquired from scanners of multiple vendors (Philips, Siemens, and GE) and models using clinically established diffusion-weighted imaging protocols, and we were unable to harmonize the acquisition protocols across centers or statistically adjust for these system-level factors. Because magnetic field strength inevitably correlates with the scanner vendor and other technical factors that can influence quantitative ADC values—including the scanner model, slice thickness, field of view, and acquisition matrix (Supplementary Table S1)—the observed differences should be interpreted as the combined effect of magnetic field strength and its correlated acquisition parameters rather than as a result of magnetic field strength alone [19,21]. Accordingly, the quantitative ADC thresholds derived here may not be directly transferable between scanners with different field strengths and protocols, and scanner-specific or harmonized acquisitions are desirable before universal ADC cutoffs are applied. Second, the time window for MRI scans was relatively wide [8]. Recent studies have reported the phenomenon of pseudonormalization, in which ADC values fluctuate following cardiac arrest [22]. Since the MRI scans in this study were performed for clinical purposes and only once per patient, we could not analyze how temporal changes in the ADC might have influenced our results. However, the timing of MRI acquisition did not differ significantly between the 1.5 T and 3.0 T groups (mean 77.4 vs. 78.3 h; *p* = 0.467; Table 1), making it unlikely that scan timing systematically confounded the comparison between field strengths. Nevertheless, future prospective studies with standardized imaging timing are warranted to minimize the potential influence of temporal ADC changes. Third, although 1354 patients were originally registered in the registry, the final cohort for analysis comprised 538 patients. In this cohort, approximately two-thirds of the patients had poor outcomes, suggesting that patients with good neurological prognoses may not have undergone MRI in actual clinical practice. Such selection bias may affect the generalizability of the results, necessitating caution in their interpretation.

## 5. Conclusions

In this study, we found that magnetic field strength (1.5 T vs. 3.0 T) may affect voxel-based quantitative ADC analysis in patients who achieved ROSC after out-of-hospital cardiac arrest and received TTM. AUC values were numerically higher at 3.0 T across all ADC indices, although this difference was statistically significant only for the PV500 index. These findings suggest a potential advantage of higher field strength rather than a definitive superiority across all evaluated metrics and support the need to establish field-strength-specific thresholds. Prospective and more rigorously controlled studies are warranted to validate these results.

## Figures and Tables

**Table 1:** Demographic characteristics of the subjects according to MRI field strength (1.5 T vs. 3.0 T).

	Total	1.5 T	3.0 T	*p*
	(n = 538)	(n = 259)	(n = 279)	
Age	56.9 ±15.5	57.6 ± 15.3	56.4 ± 15.6	0.373
Female	160 (29.7)	76 (29.3)	84 (30.1)	0.846
History				
HTN	168 (31.2)	84 (32.4)	84 (30.1)	0.561
DM	117 (21.7)	68 (26.3)	51 (18.3)	0.015
CAD	102 (19.0)	55 (21.2)	47 (16.8)	0.194
CeVD	21 (3.9)	13 (5.0)	8 (2.9)	0.783
Cardiac cause	311 (57.8)	171 (66.0)	140 (50.2)	0.019
Witnessed	380 (70.6)	212 (81.9)	168 (60.2)	<0.001
Bystander CPR	337 (62.6)	165 (63.7)	172 (61.6)	0.622
Shockable rhythm	215 (40.0)	113 (43.6)	103 (36.6)	0.094
Arrest time (min)	29.0 ± 18.1	27.8 ± 19.2	29.9 ± 16.9	0.150
MRI time (hr)	77.9 ± 13.9	77.4 ± 15.1	78.3 ± 12.7	0.467
Good outcome	195 (36.2)	105 (40.5)	90 (32.3)	0.046

Abbreviations: MRI—Magnetic resonance imaging; HTN—Hypertension; DM—Diabetes mellitus; CAD—Coronary artery disease; CeVD—Cerebrovascular disease; CPR—Cardiopulmonary resuscitation.

**Table 2:** ADC and Percent Voxel parameters by MRI field strength within outcome groups.

	Good Outcome			Poor Outcome		
	1.5 T	3.0 T	*p*	1.5 T	3.0 T	*p*
Mean ADC	819.0 (802.0–835.1)	812.0 (793.1–837.8)	0.072	672.6 (573.6–809.8)	669.1 (554.7–765.9)	0.952
PV650	6.4 (4.7–9.0)	9.1 (6.2–12.1)	0.012	44.9 (11.8–69.6)	46.4 (21.0–75.1)	0.252
PV600	3.0 (2.1–5.0)	4.4 (2.8–6.6)	<0.001	33.5 (6.0–57.9)	34.9 (12.2–64.3)	0.782
PV550	1.7 (1.0–2.7)	2.6 (1.6–4.0)	0.002	21.3 (3.2–43.2)	23.4 (7.5–51.3)	0.353
PV500	1.2 (0.5–1.8)	1.7 (1.0–2.4)	0.009	10.0 (1.8–29.2)	15.3 (4.6–35.0)	0.252

Abbreviations: ADC—Apparent diffusion coefficient; MRI—Magnetic resonance imaging; PV—Percent voxel.

**Table 3:** Prognostic performance and optimal thresholds for poor outcome prediction.

1.5 T	Cut-Off	AUC	Sensitivity	Specificity	PPV	NPV
PV650						
Youden	≥15.1	0.824	71.4 (63.6–78.4)	96.2 (90.5–99.0)	96.5 (91.3–99.0)	69.7 (61.5–77.0)
0	≥72	(0.772–0.868)	21.4 (15.2–28.8)	100.0 (96.5–100.0)	100.0 (89.4–100.0)	46.5 (39.8–53.2)
PV600						
Youden	≥7.3	0.831	72.7 (65.0–79.6)	93.3 (86.7–97.3)	94.1 (88.3–97.6)	70.0 (61.7–77.4)
0	≥67.6	(0.779–0.874)	16.8 (11.3–23.8)	100.0 (96.5–100.0)	100.0 (86.8–100.0)	45.1 (38.6–51.7)
PV550						
Youden	≥5.1	0.831	68.8 (60.9–76.0)	95.2 (89.2–98.4)	95.5 (89.8–98.5)	67.6 (59.4–75.0)
0	≥60.7	(0.779–0.874)	12.3 (7.6–18.6)	100.0 (96.5–100.0)	100.0 (82.4–100.0)	43.7 (37.4–50.3)
PV500						
Youden	≥3.2	0.821	66.2 (58.2–73.6)	95.2 (89.2–98.4)	95.3 (89.4–98.5)	65.8 (57.7–73.3)
0	≥48.3	(0.769–0.866)	7.8 (4.1–13.2)	100.0 (96.5–100.0)	100.0 (73.5–100.0)	42.5 (36.3–48.9)
**3.0 T**						
PV 650						
Youden	≥18.7	0.877	77.3 (70.6–83.0)	91.1 (83.2–96.1)	94.8 (90.0–97.7)	65.6 (56.6–73.9)
0	≥79.8	(0.833–0.913)	21.2 (15.6–27.7)	100.0 (96.0–100.0)	100.0 (91.2–100.0)	37.7 (31.5–44.1)
PV600						
Youden	≥13.0	0.889	74.1 (67.2–80.2)	96.7 (90.6–99.3)	97.9 (94.0–99.6)	64.0 (55.3–72.0)
0	≥71.4	(0.847–0.924)	18.5 (13.3–24.8)	100.0 (96.0–100.0)	100.0 (90.0–100.0)	36.9 (30.8–43.3)
PV550						
Youden	≥7.8	0.894	74.6 (67.8–80.6)	97.8 (92.2–99.7)	98.6 (95.0–99.8)	64.7 (56.1–72.7)
0	≥58.5	(0.852–0.927)	18.0 (12.8–24.2)	100.0 (96.0–100.0)	100.0 (89.4–100.0)	36.7 (30.7–43.1)
PV500						
Youden	≥4.5	0.895	75.1 (68.3–81.1)	95.6 (89.0–98.8)	97.3 (93.1–99.3)	64.7 (55.9–72.7)
0	≥43.7	(0.853–0.928)	17.5 (12.3–23.6)	100.0 (96.0–100.0)	100.0 (89.4–100.0)	36.6 (30.6–42.9)

Abbreviations: AUC—Area under curve; PPV—Positive predictive value; NPV—Negative predictive value; PV—Percent voxel.

**Table 4:** Comparison of ROC curve by magnetic field strength.

	1.5 T		3.0 T		
	AUC	Standard Error	AUC	Standard Error	*p*
PV500	0.821	0.0263	0.895	0.0194	0.025
PV550	0.831	0.0260	0.894	0.0194	0.051
PV600	0.831	0.0263	0.889	0.0198	0.074
PV650	0.824	0.0270	0.877	0.0208	0.120

Abbreviations: AUC—Area under curve; PV—Percent voxel.

## Data Availability

The dataset generated and analyzed during this study is available from the corresponding author upon reasonable request.

## Supplementary Materials

The following supporting information can be downloaded at https://www.mdpi.com/article/10.3390/jcm15145411/s1, Table S1: MRI scanners and diffusion (ADC) acquisition parameters by center and field strength.
