# Supplementary material for: Impact of Magnetic Field Strength (1.5 T vs. 3.0 T) on the Prognostic Value of Quantitative ADC Indices in Patients After Out-of-Hospital Cardiac Arrest: A Prospective Multicenter Observational Study in Korea (The KORHN-PRO Registry)
Source: J Clin Med. 2026 Jul 10;15(14):5411. doi: 10.3390/jcm15145411 (PMC13412463; doi:10.3390/jcm15145411)
Supplement: Supplementary file 1 [file jcm-15-05411-s001.zip › jcm-4378567-supplementary.pdf]

Supplementary Table S1. MRI scanners and diffusion (ADC) acquisition parameters by center and field strength

Center	Unit	Vendor	Model	Field (T)	Max gradient (mT/m)	Slew rate (T/m/s)	TR (ms)	TE (ms)	Slice thickness (mm)	Slice gap (mm)	Acquisition matrix (PE)	FOV (mm)	Matrix	Coil
Center 1	Philips	Philips	Ingenia	3	45	200	2360.6–4829.9	64.8–97.7	2.5–5	3.5–6.5	124–133	240 × 240	256 × 256	MULTI COIL
Center 2	Siemens	Siemens	Avanto	1.5	45	200	3000–7733	86–87	3–5	3–7	192	240 × 240	128 × 128	d:HE1-4; d:HE1-4;NE2; d:HE3,4;NE2
Center 3	Philips	Philips	Achieva	1.5	33	160	5182	76	4	4	120	230 × 230	128 × 128	SENSE-Head-8
Center 3	Siemens	Siemens	Verio	3	45	200	4100–9541	61–106	5	6	128–192	230 × 230	128 × 128	HeadMatrix
Center 4	Siemens 1	Siemens	Avanto	1.5	45	200	3500–3600	89	5	6.5	180	230 × 230	192 × 192	T:HE1-4;NE1,2; T:HE1-4;NE2
Center 4	Siemens 2	Siemens	Verio	3	45	200	6000–6800	100	5	6.5	192	230 × 230	192 × 192	T:HEA;HEP; T:HEA;HEP;NE1,2; T:HEA;HEP;NE2
Center 5	GE	GE	DISCOVERY MR750	3	50	200	3661–4000	63–65.9	5	6.5–8	160	210 × 210<br>220 × 220	256 × 256	HNS Head
Center 6	Philips 1	Philips	Achieva	1.5	33	160	2733.8–3287.1	65.1–83.1	5	6.5	128–136	230 × 230	256 × 256	SENSE-Head-8; SENSE_HEAD_8
Center 6	Philips 2	Philips	Ingenia	3	45	200	3065.5–3407.4	85.2–87.1	5	6.5	192–210	240 × 240	288 × 288	MULTI COIL
Center 6	Siemens	Siemens	Verio	3	45	200	5800–6700	97	5	6–6.5	173	230 × 230	256 × 256	T:HEA;HEP;NE2
Center 7	Philips 1	Philips	Achieva	1.5	33	160	4207.6–8422.1	53.4–53.9	3	3	126	240 × 240	256 × 256	SENSE-Head-8; SENSE-NV-16
Center 7	Philips 2	Philips	Achieva	3	40	200	3000	44–44.5	3	3.2–3.5	130	240 × 240	256 × 256	SENSE-Head-8; SENSE-NV-16
Center 8	Siemens	Siemens	Skyra	3	45	200	6800–7510	64–72	4	4.4	158–160	240 × 240	256 × 256	HeadNeck_20
Center 9	Philips 1	Philips	Achieva	3	40	200	3500	47.2–47.5	5	6–6.16	126	240 × 240	512 × 512	SENSE_HEAD_8; SENSE_NEURO_VASC
Center 9	Philips 2	Philips	Ingenia	3	45	200	2723.5–4772.8	83.5–87.2	5	6–6.9	90–126	220 × 220	512 × 512	MULTI COIL
Center 10	Philips	Philips	Achieva	3	40	200	5206.5–5261.5	73.8–76	5	6	112	220 × 220<br>240 × 240	256 × 256	SENSE-Head-8

Center 11	GE	GE	DISCOVERY MR750w	3	50	200	6949	70.7	5	5	128	260 × 260	256 × 256	Head 24
Center 12	Siemens	Siemens	Avanto	1.5	45	200	4000–4400	84–95	5	6–7	120–192	230 × 212	240 × 260	T:HE1-4; T:HE1-4;NE2
Center 13	Philips	Philips	Achieva dStream	1.5	33	160	4278.2–4290.6	60	3	3	128	240 × 240	256 × 256	MULTI COIL
Center 14	Philips	Philips	Achieva	3	40	200	2750.9–12000	65.3–86.5	3–5	3–6	128–158	230 × 230 240 × 240	256 × 256	Dual coil; MULTI COIL; SENSE-Head-8
Center 15	Philips	Philips	Ingenia	3	45	200	12000	71.6–72.7	3	3	128	240 × 240	256 × 256	MULTI COIL
Center 16	Siemens	Siemens	Prisma_fit	3	80	200	4380–5570	56–82	4–5	4.8–6	164–240	220 × 220	256 × 256	N/A
Center 17	Siemens	Siemens	Skyra	3	45	200	6520–7740	57–65	4	4.8	160–164	220 × 220	384 × 384	N/A
Center 18	GE	GE	SIGNA Architect	3	44	200	3890–5184	70.1–76.2	3	N/A	128	240 × 240	256 × 256	Head 34; Head 48

- *Field strength is taken from the DICOM MagneticFieldStrength tag, not the protocol name.*
- *Within a center, multiple physical scanners are distinguished by a number after the vendor (e.g., Siemens 1, Siemens 2).*
- *Acquisition parameters (TR, TE, slice thickness, gap, matrix) are read from the DICOM headers; cells give the range across studies (single value if constant).*
- *Max gradient and slew rate are typical manufacturer specifications for each model (not stored in DICOM) and may vary with the installed gradient configuration.*
- *All diffusion-weighted acquisitions used b-values of 0 and 1000 s/mm².*
- *FOV (mm) is the in-plane reconstructed field of view (frequency × phase) read from representative diffusion DICOMs (one to two scans per center); values give the range across sampled scans. N/A = no representative scan available for that model (e.g., Skyra, MR750w).*
